# Immunosurveillance of *Candida albicans* commensalism by the adaptive immune system

**DOI:** 10.1038/s41385-022-00536-5

**Published:** 2022-07-01

**Authors:** Marc Swidergall, Salomé LeibundGut-Landmann

**Affiliations:** 1 Division of Infectious Diseases, Harbor-UCLA Medical Center, Torrance, CA, USA; 2 The Lundquist Institute for Biomedical Innovation at Harbor-UCLA Medical Center, Torrance, CA, USA; 3 David Geffen School of Medicine at UCLA, Los Angeles, CA, USA; 4 Section of Immunology, Vetsuisse Faculty, University of Zürich, Zürich, Switzerland; 5 Institute of Experimental Immunology, University of Zürich, Zürich, Switzerland

## Abstract

The fungal microbiota (mycobiota) is an integral part of the microbial community colonizing the body surfaces and is involved in many key aspects of human physiology, while an imbalance of the fungal communities, termed fungal dysbiosis, has been described in pathologies ranging from infections to inflammatory bowel disease. Commensal organisms, such as the fungus *Candida albicans*, induce antigen-specific immune responses that maintain immune homeostasis. Adaptive immune mechanisms are vital in this process, while deficiencies in adaptive immunity are linked to fungal infections We start to understand the mechanisms by which a shift in mycobiota composition, in particular in *C. albicans* abundance, is linked to immunopathological conditions. This review discusses the mechanisms that ensure continuous immunosurveillance of *C. albicans* during mucosal colonization, how these protective adaptive immune responses can also promote immunopathology, and highlight therapeutic advances against *C.* albicans-associated disease.

## Introduction

Fungi are an integral part of the human microbiota interacting with the immune system and affecting human physiology. The human mycobiota is composed of 390 different fungal species belonging to the phyla *Ascomycota, Basidiomycota,* and *Zygomycota*[1], which are required for microbial community structure, metabolic function, and immune priming[2, 3, 4]. *Candida* species such as *Candida albicans* are commonly found on the oral, gastrointestinal, and vaginal mucosae. Besides its host-beneficial effects protecting the host from various microbial insults, *C. albicans* can itself become pathogenic and cause diverse pathologies in immunocompetent and immunocompromised individuals. *Candida*-mediated disorders range from mild superficial infections such as neonatal thrush to extensive and/or recurrent infections of the esophageal or vaginal tract that can cause significant morbidity[5]. Translocation of *Candida* across epithelia and fungal dissemination through the bloodstream can finally result in life-threatening systemic manifestations, which are responsible for several hundred thousand annual deaths worldwide[6]. In addition, *C. albicans* is also associated with inflammatory not primarily infectious diseases, such inflammatory bowel disease (IBD)[7]. Given the continuous presence of *C. albicans* in barrier tissues and their significant pathogenic potential, tight control of the fungus at the host interface is a prerequisite of homeostasis. The adaptive immune system plays a key role in providing long-lasting defence against uncontrolled fungal growth, acquisition of virulence traits, and invasion of normally sterile tissues.

Evidence for a protective role of T cells against *C. albicans* is provided by individuals displaying an enhanced susceptibility for mucocutaneous candidiasis due to acquired or inherited defects in the frequency, activation, or function of CD4^+^ T cells, especially those producing IL-17[8, 9]. Although historically, the evidence for antibodies contributing to fungal control was sparse, recent studies highlight the relevance of antibody-mediated immunity in maintaining *C. albicans* commensalism[10, 11, 12]. Key hallmarks of cellular and humoral adaptive immunity are the antigen-specificity, which accounts for the directionality of the response, the requirement for lymphocytes to undergo tightly regulated differentiation and polarization processes, which generate highly specialized and qualitatively distinct responses, and the capacity to form long-lasting memory.

Here, we review the current understanding of T- and B-cell mediated antifungal defence in barrier tissues, discuss how these normally protective immune mechanisms can also promote immunopathology under certain conditions, and highlight outstanding challenges that still impede harnessing our knowledge for preventative & therapeutic advances against *Candida*-associated (infectious and non-infectious) disorders.

## T cell-mediated immunity against *C. albicans*

*C. albicans*-responsive CD4^+^ T cells are primed in all healthy individuals as a consequence of their constant exposure to the fungus[13, 14]. They are characterized predominantly by a Th17 profile producing IL-17A, IL-17F and IL-22, with a minor fraction belonging to the Th1 or Th2 subsets[15]. While most studies assessing human antifungal T cells focused on circulating T cells due to their easy accessibility in the blood, *C. albicans*-specific Th17 cells have also been evidenced in the healthy skin[16]. Similarly, in experimental models of oropharyngeal candidiasis (OPC) and epicutaneous candidiasis, *C. albicans*-specific T cells with a selective Th17 phenotype are detected in the colonized epithelial tissue within a week of infection[17, 18]. In addition to Th17 cells, innate-like T cells and TCR-negative innate lymphoid cells (ILCs) also contribute to the overall IL-17 production in mice where these IL-17 producers rapidly accumulate in response to the primary exposure of the murine host with the fungus, especially in response to virulent strains of *C. albicans,* which only transiently colonize mice[19, 20, 21, 22]. The diverse cellular subsets appear to play redundant roles[19], with γδ T cells being particularly prominent/important during cutaneous experimental candidiasis[22].

The signals driving polarization of *C. albicans*-specific Th17 cells including IL-23, IL-6 and IL-1 contribute also to the activation of innate-like T cells and ILCs[19, 21, 23, 24]. Different dendritic cell subsets have been implicated at the interface between the fungus and IL-17-production in the initiation of the response depending on the IL-17-producing cellular subset and the tissue compartment (skin vs. mucosa)[23, 25, 26]. In addition, neurons (in the skin) can sense the fungus and link fungal recognition to the induction of IL-23 by dermal DCs[22]. Moreover, the cellular damage caused by the fungal peptide toxin candidalysin, which is secreted by high virulent strain of *C. albicans*, contributes to IL-1 release for rapid IL-17 induction by innate-like T cells[21].

The continuous presence of *C. albicans* during commensalism requires a long-lived response for maintenance of homeostasis over time. As such, *C. albicans*-reactive Th17 in humans exhibit a memory phenotype[13]. Within barrier tissues, they express markers characteristic of tissue-resident memory T (T_RM_) cells[16]. This has also been reproduced in experimental mice that have been persistently colonized with *C. albicans* to closely mimic the situation in humans[18].

**Fig. 1:**
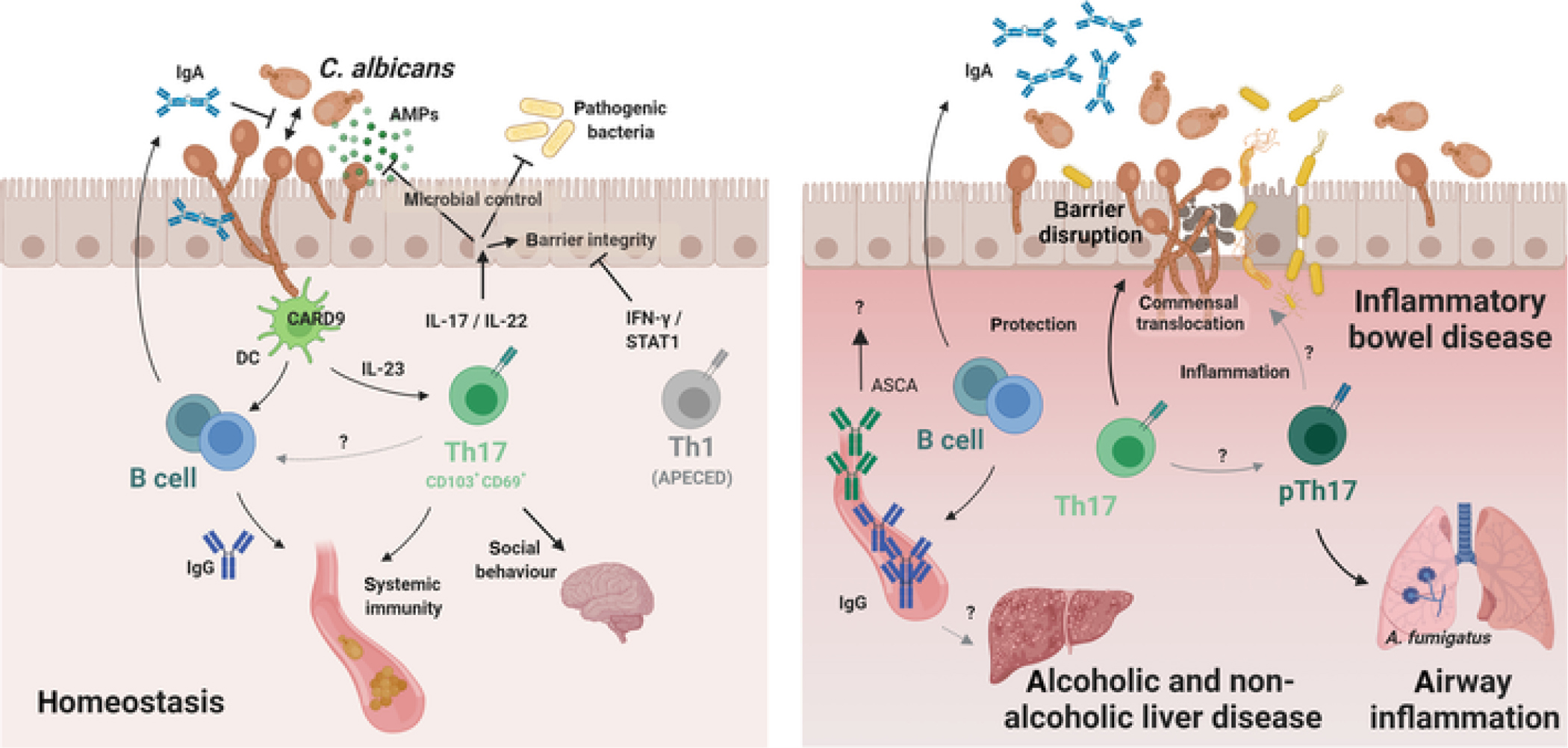
Adaptive immunity against *C. albicans* in health and disease. **(Left).** Homeostatic immunity against *C. albicans* is maintained by cellular and humoral adaptive immunity. *C. albicans* drives fungus-specific and tissue-resident Th17 cells. IL-17 and IL-22 contribute to fungal control by promoting antimicrobial and barrier functions of the epithelium, the latter of which is counteracted by overt IFN-γ/STAT1-signalling. Antifungal Th17 immunity can also promote barrier defence against heterologous infections, both locally and systemically, and modulate social behaviour. Mucosal IgA targets fungal virulence determinants to repress *C. albicans* pathogenicity. *C. albicans*-specific IgG contributes to systemic antifungal immunity. **(Right)**. Enhanced *C. albicans*-specific T cell and antibody responses have been observed in patients with gut barrier defects as in IBD. Whether IgA and Th17 cells act in a host-protective manner or whether antifungal T cells adopt features of pathogenic Th17 cells in the inflamed gut remains unclear. During airway inflammation, *C. albicans*-specific pathogenic Th17 cross-react with *A. fumigatus.* Enhanced *C. albicans*-reactive IgG antibodies have been associated with alcoholic and non-alcoholic liver disease, albeit their role in disease pathogenesis remains to be determined. See text for more details. The figure was generated with BioRender.com.

Accumulation of *C. albicans*-specific Th17 during commensalism depends on cognate antigen presentation and on Card9-dependent signals including IL-23[18, 27], but overall remains not well understood. The initiation and maintenance of the antifungal T cell response represent separate processes characterized by different signal requirements. Overall, the immunosurveillance response is uncoupled from inflammation, in line with the notion that inflammation is not compatible with homeostasis and consistent with what was also shown for the homeostatic T cell response against commensal bacteria[28]. The tonic signal controlling long-term maintenance of antifungal T cells during commensalism in colonized tissue remains to be determined. Rapid decline of the antifungal T cell population following antimycotic-mediated removal of the fungus indicates that maintenance of the homeostatic Th17 response depends on the continuous presence of the fungus[18]. This is reminiscent of the situation of other commensal- and virus-specific tissue-resident CD4+ T cells responses[29, 30]. The dependence on fungal persistence may reflect the requirement for continuous antigen recognition by commensal-specific Th17 cells, as shown to be the case for homeostatic Th1 cells in the skin, which depend on keratinocyte-intrinsic MHC-II[31]. Alternatively, fungal persistence might induce cytokine signals for homeostatic T cell survival, proliferation and/or renewal or modulate the microenvironment in another way to favour T cell persistence. The observed dependence of the *C. albicans*-specific T cell response on the continuous presence of the fungus contradicts the paradigm of immunological memory. Therefore, clarification of the relationship between homeostatic T cells providing immunosurveillance of commensals and memory T cells that protect against recurring infections awaits further investigations. Taken together, long-lived Th17 cells residing in the colonized tissue are the most notable ones of the diverse lymphoid cells that contribute to the overall IL-17 response for immunosurveillance of commensalism and homeostasis (Fig. 1A), although their identity and regulation remains to be defined in more detail.

*C. albicans*-specific Th17 cells act locally in the colonized tissue to keep homeostasis under control and prevent fungal overgrowth and tissue invasion by strengthening the antimicrobial effector functions and the barrier integrity of the epithelium. Reinforcement of the barrier function and protection from intestinal injury in the gut was attributed to IL-22, which is produced by antifungal Th17 cells in addition to IL-17A and IL-17F[32]. Of note, the T cell response mounted in response to fungal colonization enhances resistance not only against the mycobiota itself but also against bacterial infections as shown in experimental models with *C. difficile* and *C. rodentium*[32, 33].

While an impairment of the IL-17/IL-22 pathway predisposes individuals to mucocutaneous candidiasis, the same fungal infection also manifests in individuals with an intact antifungal Th17 response but a dysregulated balance between type 17 and type 1 immunity. As such, APECED patients suffering from chronic mucocutaneous candidiasis (CMC) exhibit exacerbated type 1 responses while type 17 immune responses are intact[34]. *Aire*-deficient mice recapitulating the situation in APECED patients revealed that IFN-γ-driven interferonopathy drives pronounced epithelial barrier defects, which underlie the mucosal fungal susceptibility[34]. Barrier-protective effects of type 17 immunity can thus be overridden by aberrant IFN-γ/STAT1 responses that promote epithelial cell death and barrier disruption.

Beyond the local host-protective effects, *C. albicans*-induced Th17 immunity has also been reported to mediate systemic antimicrobial effects[35, 36] including heterologous protection from systemic *S. aureus*[35]. Mechanistically, it was proposed that Th17 primed locally in the gut promote systemic neutrophil activation to enhance protection against an intravenous challenge[35]. Moreover, the mycobiota can impact physiological processes beyond host protection. As such, fungal gut colonization was recently proposed to promote social behaviour in mice[32]. The neuromodulatory role of mucosa-associated fungi occurs in an IL-17-dependent manner with neuronal cells acting as direct targets of IL-17[32]. This expands the previously noted effects of IL-17 on social behaviour[37, 38, 39, 40] and provides a mechanistic basis for the reported associations of fungal dysbiosis and neuropsychiatric conditions in humans[41, 42].

During homeostasis, *C. albicans*-specific Th17 cells are not associated with inflammatory processes despite their continuous engagement by the commensal fungus and the proinflammatory potential of IL-17. This is reminiscent of homeostatic T cells directed against bacteria in the gut, which display a metabolic profile characteristic of resting memory cells[29]. Whether immunoregulatory processes contribute to stable homeostasis by actively preventing plasticity of *C. albicans*-specific homeostatic Th17 cells towards pathogenicity (see below) remains currently unclear with no evidence for a contribution of regulatory T cells or IL-10[43]. Moreover, the antifungal effects of Th17 cells during fungal commensalism are not accompanied by an accumulation of neutrophils in the colonized tissue, which would be incompatible with homeostasis, even though neutrophil-recruiting chemokines are stimulated by IL-17 under some conditions. Examples of mucocutaneous *C. albicans* infections characterized by a strong neutrophil response in the infected tissue are those from experimental cutaneous and oropharyngeal infections with highly virulent strains of *C. albicans*[44, 45], where the inflammatory response is an acute and direct reaction to fungal virulence factors and release of keratinocyte-derived alarmins such as IL-1α[21], and is largely independently of the IL-17 pathway[45]. In conclusion, the homeostatic role of IL-17 is non-inflammatory and expands beyond microbial control, and it may be mediated by mechanisms beyond strengthening antimicrobial barrier functions of the epithelium (Fig. 1A).

## Antibody-mediated immunity against *C. albicans*

Antibodies play a vital role in homeostasis and protective immunity at mucosal sites, as well as during systemic infections[46, 47], and can be divided into five isotypes, which operate in distinct places and have distinct effector functions[48]. Antibody effector functions have classically been studied in the context of pathogenic infections[49]. However, it is apparent that luminal and serum antibodies bind members of the microbiota[50].

The fungal cell wall contains various pattern-associated molecular patterns (PAMPs), such as β-glucans, for innate immune recognition[51, 52, 53, 54]. However, the *C. albicans* cell wall and secretory proteins are also a significant source of antigens[55]. During the course of an immune response, depending on the nature of the eliciting antigen and its entry mode, class-switch recombination (CSR) replaces immunoglobulin constant regions for the isotype that can best protect against commensal or pathogenic microorganism[56]. As a result, mature B cells express antibodies of the IgA, IgG, or IgE classes that differ in effector functions without altering the specificity for the immunizing antigen[57]. Intestinal *C. albicans* colonization induces distal CSR and B cell expansion in germinal centers (GC) to induce high-affinity IgG. CSR and GC-B cell expansion is controlled by CARD9 and CX3CR1^+^ mononuclear phagocytes (MNPs)[58].Antifungal IgG responses are reduced in patients with polymorphisms in the coding region of the *CX3CR1* or *CARD9* genes[58, 59], in whom CD has been described[60, 61, 62, 63]. Furthermore, CARD9-deficient patients manifest fungal-specific infection susceptibility, predominantly in the central nervous system by *C. albicans*[64, 65].

A significant portion of *C. albicans* is recognized by systemic antifungal IgG antibodies in humans and mice, where IgG3 binds the largest fraction of intestinal fungi[58]. IgG3 Abs are potent mediators of effector functions, including enhanced Ab-mediated cellular cytotoxicity, opsonophagocytosis, complement activation, and neutralization, compared with other IgG subclasses[66]. Accordingly, mucosal fungal colonization induces distal humoral immunity and systemic protection against invasive candidiasis.

Cross-reactive antibody responses provide protective immunity to related pathogens or antigenic variants in natural epidemiology. In this line, intestinal-induced anti-*C. albicans* IgG protects against systemic infection with the emerging drug-resistant fungus *C. auris*[58]. However, IgG isotypes generated by intestinal *C. albicans* colonization are not cross-reactive with environmental, food-derived, or skin-resident fungi such as *Saccharomyces, Aspergillus*, and *Malassezia* spp[58].

IgA is the dominant isotype at mucosal barriers[67], and predominantly induced in response to colonization with commensal organism to maintain homeostasis[68]. Within the mucosa, commensal microorganism are coated by low-affinity and antigen-specific secretory IgA (SIgA)[69]. Furthermore, IgA serves as the first line of defense in protecting the epithelium, from toxins or potential pathogenic microorganism overgrowth[68]. While immune exclusion is a dominant IgA effector mechanism[68], IgA is able to enchain pathogens, thereby preventing organism separation after replication resulting in clumping[70]. Accordingly, mucosal *C. albicans* colonization induces IgA[10, 11, 12] to reduce fungal-associated virulence attributes[11]. IgA binding to *C. albicans* prevents adhesion, invasion, and damage of epithelial cells[11, 21] resulting in reduced inflammation[11]. In particular, IgA binds to hyphae-associated virulence factors, such as *C. albicans* adhesins or the toxin candidalysin[10, 12]. Consequently, IgA binding controls commensal homeostasis by eliminating *C. albicans*-associated virulence traits, while the absence of IgA results in mucosal dysbiosis, *C. albicans* overgrowth, and pathology[10, 11, 12]. Associated pathology is observed in patients with CD, in whom reduced virulence factor targeting SIgA have been described[10], and in NDV3 vaccinated mice, which generate IgA against the adhesion Als3 and have reduced pathology during DSS-induced colitis[12]. Together, by modulating the *C. albicans* virulence IgA-dependent immunity favours fungal commensalism and homeostasis at mucosal barriers.

Most antibody-secreting cells (ASCs) in mucosal tissues produce IgA[72]. Oral mucosal *C. albicans* colonization increases CD19^+^ CD138^−^ B cell, plasmablast, and plasma cell enrichment in the mucosa[11], while intestinal colonization increases the IgA^+^ frequency and strong IgA CSR in the PP B cell compartment associated with high-affinity antibody responses[10]. These IgA responses are mediated through innate immune interaction with intestinal CD11c^+^CD11b^+^CD103^+^ dendritic cells and CX3CR1^+^ MNPs[10]. Trafficking of IgA ASCs is regulated by a combination of chemokine receptors as well as integrins[46]. Recent evidence points at a role for gut-educated IgA ASCs in modulating antifungal immune responses outside of mucosal tissues. During homeostasis, meninges, the membranes surrounding the brain and spinal cord, contain gut-derived and commensal-specific IgA ASCs[73]. Mice lacking IgA or with a selective loss of meningeal IgA plasma cells exhibit reduced protection against *C. albicans* and is associated with increased fungal invasion of the brain[74].

SIgA can cross-react with a diverse fraction of the microbiota by canonical Fab-dependent and non-canonical carbohydrate-dependent binding[75]. Oral mucosal colonization with *C. albicans* increases total levels of cross-specific IgAs against the common oral commensal *Streptococcus oralis*[11] suggesting that fungal colonization and cross-reactive IgA responses shape the microbial community.

B cells have diverse antibody-independent functions, such as production of regulatory and pro-inflammatory cytokines, as well as antigen presentation and T cell stimulation[76]. Human B cells can present fungal antigens to T cells in an HLA-DR–restricted manner, provide critical costimulatory signals through CD80 and CD86, and induce Th17 cell differentiation through an IL-6-dependent mechanism[77], while secretion of IL-6 depends on hyphal stimulation and is mediated by MyD88[78].

Collectively, mucosal *C. albicans* colonization induces a variety of antibody responses, which not only restricts fungal overgrowth, but also limit virulence, locally and systematically (Fig. 1A).

## Effects of the adaptive antifungal immunity on the intraspecies diversity of *C. albicans*

*C. albicans* displays high intraspecies diversity, notably variations in the distribution of heterozygous polymorphisms along the genome[79, 80, 81]. Genetically distinct isolates differ in their phenotype such as the degree of filamentation, expression of virulence factors, the induction of epithelial cell damage or the degree of inflammation-induced at the host interface. These phenotypic variations translate in differential outcomes of the interaction with the host as evidenced when probing genetically distinct isolates in model hosts which exclude any inter-individual differences on the host side prior to infection[20, 79, 82, 83, 84]. From studying large sets of isolates it became clear that the high-virulent strain SC5314, which is broadly used in in vitro and in vivo experimental studies, is a poor colonizer of mucosal surfaces in immunocompetent hosts[20, 85] and thereby represents rather an outlier than the norm within the species of *C. albicans*[20]. Despite striking differences between high- and low-virulent isolates in their efficiency to colonize mucosal surfaces, the adaptive immune response mounted in response to oral colonization is surprisingly comparable with strong T cell and antibody responses induced against widely differing isolates[11, 20]. This is consistent with the observation that all human individuals mount a *C. albicans*-specific T cell and antibody response, irrespective of the isolate that they are colonized with[10, 12, 13, 14]. This suggests that *C. albicans* virulence and immunogenicity are largely uncoupled for T cell priming in the oral mucosa and that antigens, as well as PRR ligands mediating dendritic cell activation for efficient lymphocyte priming, are more strongly conserved between isolates than the expression of virulence factors. PAMP exposure by *C. albicans* seems to depend on the body site of isolation[86]. Moreover, because *C. albicans* actively masks PAMPS in response to its environment to reduce immune recognition[87], niche-specific colonization and consequently PAMP exposure may influence immune activation and thereby favour commensalism.

Adaptive immunity can modulate the pathogenicity of *C. albicans*[10, 12]. Thus, variations in the activity of antibody and/or T cell effector functions over time may generate a spectrum of phenotypes within an individual. If different phenotypes become epigenetically stabilized through DNA methylation[88] or genetically fixed over time through (micro)evolutionary processes, this might contribute to the generation of genetic diversity. Indeed, *C. albicans* also undergoes genomic rearrangement during oral infection of mice[89]. Growth in the oral mucosa of mice selects for trisomy of chromosome 6, resulting in a commensal-like phenotype[90]. Importantly, *C. albicans* within-host diversity was also observed in humans and appears common in the context of commensalism as demonstrated by recent genome sequencing of isolates collected from single healthy individuals, which showed that they differed by numerous single nucleotide polymorphisms and short-range loss-of-heterozygosity (LOH)[91]. *C. albicans* genomic and epigenetic variations can facilitate adaptation to environmental changes and improve the persistence of the fungus in various host niches[92]. When considering inter-individual variations in the host adaptive immune activity, including those arising from to the increasing population of immunocompromised individuals, lineage diversification resulting from the close contact of *C. albicans* with the human host is an important driver of the genetic diversity observed within the species of. *C. albicans*[79, 80, 81]. Therefore, mutual adaptations of *C. albicans* and its mammalian host shape the outcome of their interaction both, at an individual’s level as well as at a population scale.

## Immunopathological consequences of adaptive immunity against *C. albicans*

While *C. albicans* is a common inhabitant of mucosal tissues in healthy individuals, fungal dysbiosis has been associated with diverse human diseases, including inflammatory disorders of colonized tissues such as gut, oral cavity, and skin, as well as pathologies in distant organs, including liver and lung. Whether and how the development and/or progression of pathology is causally linked with the observed shift in mycobiota composition remains often unclear and in many cases, the connection appears to be rather unspecific relying on innate mechanisms. However, several examples have recently emerged, where adaptive immune responses directed against *C. albicans* have been implicated in disease pathogenesis, comprising both T cell and antibody-mediated scenarios (Fig. 1B).

### T cell-mediated immunopathologies

*C. albicans* (as well as other commensal fungi) are potent inducers of type-17 polarized responses, and IL-17 is implicated in the aetiology of many inflammatory disorders. As such, re-activation of mycobiota-specific Th17 cells can convey host-adverse effects and significantly aggravate tissue inflammation. In an experimental model of psoriasis, epicutaneous association of the murine skin with *C. albicans* was shown to exacerbates psoriaform skin inflammation in a Th17-dependent manner[93], in line with the observed enrichment of *Candida-species* in lesional skin of psoriasis patients[94] and the positive response of defined psoriatic patients to antifungals[95]. Similar results have been obtained with other skin commensal yeasts and in other models of skin inflammation[93, 96]

Dysbiosis in Crohn’s disease (CD) patients is characterized by an increase in *C. albicans* and other *Candida* species[97, 98, 99, 100, 101, 102]. Accordingly, *C. albicans*-specific Th17 cells are increased in the blood of CD patients[14], presumably as a consequence of enhanced microbial translocation in the inflamed and barrier-disrupted gut, and their frequencies correlate with the faecal abundance of *C. albicans* in intensive care unit patients[35]. Most recently, the pathogenicity of gut-colonizing *C. albicans* isolates was identified as a decisive factor for enhanced inflammation in IBD patients[103]. In light of the harmful rather than protective effects of IL-17 blockade in inflammatory bowel disease (IBD) patients[104], the role of *C albicans*-specific Th17 cells in CD pathogenesis remains controversial. Whether *C. albicans*-specific T cells acquire pathogenicity in the context of colitis and which factors would drive such functional plasticity remains unclear. Serum amyloid A (SAA) is one factor proposed to promote the development of colitogenic Th17 cells in the gut in a *C. albicans*-independent context[105]. Alternatively, *C. albicans*-specific Th17 may exert host-protective effects in the inflamed gut, presumably via strengthening barrier repair functions[106] or by targeting translocated microbes. This notion is supported by the observation that *C. albicans* can enhance protection from colitis in experimental models, although T cell-dependence of this effect was not elaborated in all cases[3, 59, 107].

*C. albicans*-specific Th17 cells are not restricted to colonized epithelia but can also be found systemically and in distant organs such as the lung, where *C. albicans* is not a common resident[108]. Th17 cells are strongly expanded in the inflamed lung of chronic obstructive pulmonary disease (COPD) and asthma patients, which are frequently sensitized to *Aspergillus*[14]. In fact, Th17 cells responding to *A. fumigatus* also responded to *C. albicans*[14]. Thereby, normally protective intestinal Th17 responses mounted against *C. albicans* are directly linked to lung pathologies caused by airborne fungi, albeit without signs of plasticity towards Th2 as observed in the skin[109]. While bystander activation via T cell receptor-independent mechanisms cannot be excluded for the dual responsiveness of these cells, cross-reactivity has emerged as a common mechanism for the modulation of immune responses by the microbiota[110]. Of note, the majority of Th17 cells responsive against widely diverse fungal species are cross-reactive to *C. albicans* suggesting broad modulation of human anti-fungal Th17 responses by a single fungal species[14]. Antigen-specificity is a key hallmark of adaptive immunity. Only a few fungal antigens have been identified so far[14, 15, 111]. Knowing the antigenic epitopes recognized by antifungal T cells is an important basis for understanding the mechanism that underlies the cross-reactivity of *C. albicans*-specific T cells. Towards that goal, several protein targets of cross-reactive T cells have been identified in *A. fumigatus* proteins displaying sequence similarity with their homologues in *C. albicans*[14]. Identification of *C. albicans* epitopes further offers the possibility to track fungal-specific T cells in the endogenous repertoire in an antigen-specific manner by means of MHC tetramers, providing a high-resolution approach to dissect immune responses directed against the mycobiota in health and disease, and it may reveal new targets for diagnostics and therapy.

### Antibody-mediated immunopathologies

Antibodies against fungal cell wall components and embedded proteins have been associated with various pathological conditions. Anti-*Saccharomyces cerevisiae* antibodies (ASCA), which target cell-wall components in *Saccharomyces* and *Candida* species were found elevated in the serum (IgG) and intestinal lumen (IgA) of CD patients[10, 112]. High ASCA levels are a consequence of intestinal injury suggesting that ASCA is not an epiphenomenon but is probably due to a cross-reactivity with antigens involved in the immunopathology of CD[113]. Mannans from other yeasts, such as *C. albicans*[114] can cross-react with ASCA suggesting that other yeast may induce ASCA-associated diseases[115]. In fact, in a mouse model of dextran sodium sulfate (DSS)-induced colitis *C. albicans* colonization generates ASCA and promotes inflammation[116]. In this context, systemic ASCA develops consistently in patients with CD[117, 118], in whom fungal dysbiosis and overgrowth of *Candida* species have been described[101, 119]. Furthermore, an increase in *Candida* abundance and serum ASCA generation was shown in patients with alcohol-associated liver disease[120, 121]. ASCA levels correlated with disease severity and increased mortality[122], and were reversed upon 2 weeks of alcohol abstinence[120]. Similarly, anti-*C. albicans* antibodies were also elevated in non-alcoholic fatty liver disease patients and correlated with the degree of fibrosis[123]. Antifungal therapy prevented ethanol-induced liver disease in mice[121], although it remained open whether this effect was mediated in an antibody-dependent manner and whether fungal dysbiosis and *C. albicans* overgrowth increased inflammatory responses independently of ASCA to promote pathological conditions.

In autoimmune diseases, autoantibodies (auto-Abs) often develop before any clinical symptoms can be detected[124]. While fungal dysbiosis has not been associated with the generation of auto-Abs, antibodies against cytokines and chemokines involved in mucosal antifungal immunity were proposed to predispose for *C. albicans* outgrowth and infection[125]. High antibodies titers against IL-17A, IL-17F, and IL-22 can be found in patients with CMC and autoimmune polyendocrine syndrome type I[126, 127]. However, some patients with persistent IL-17 auto-Abs lack CMC, and some patients with CMC lack auto-Abs[128, 129] suggesting the existence of CMC subtypes across a spectrum of impaired type 17 immunity, whereby in patients with an intact IL-17 pathway CMC was shown to develop as a consequence of immunopathology caused by excessive type 1 inflammation[34, 130].

## Vaccines against *C. albicans*

Vaccines are among the most successful public health interventions[131] resting on the principle of immune memory, whereby a secondary challenge induces an enhanced immune response against a previously encountered pathogen. The fact that *C. albicans* has coevolved with humans for at least 2,000 years[132], that this fungus is a lifelong inhabitant of mucosal surfaces[133], and every colonized individual harbour a memory response directed against *C. albicans*[10, 12, 13, 14], presents a number of conceptual and technical challenges in the development of an anti-*C. albicans* vaccine. Furthermore, rather than acquisition, *C. albicans* infections result from translocation or fungal outgrowth[6, 134, 135] implying that anti-*C. albicans* vaccines should target specific virulence factors to diminish pathogenic attributes. The use of the N-terminal region of *C. albicans* agglutinin like sequence 3 protein (Als3) is the most promising and advanced vaccination strategy yet tested. This vaccine, referred to as NDV-3, induces T cell[136], IgG, and IgA[12, 137] responses in mice and humans. NDV-3 vaccinated mice are protected against systemic and mucosal *C. albicans* infection[136, 138, 139, 140], while inducing cross-reactive protective responses against *C. auris* and *Staphylococcus aureus* in mice[136, 141]. Furthermore, NDV-3 vaccination prevents *C. albicans*-associated damage in mice with colitis by inducing fungal adhesin-specific IgA responses[12]. Importantly, NDV-3 has been shown to be safe and efficacious in a clinical trial against recurrent vulvovaginal candidiasis[142]. A different study uses the recombinant *C. albicans* secreted aspartyl proteinase 2 (Sap2) protein[143]. Sap2 plays an important role in fungal pathogenesis by degrading host proteins at mucosal sites and inactivate complement components[144, 145, 146]. Vaccination with Sap2 results in the generation of antigen specific protective antibodies[147], which cross-react with different members of the Sap family[148]. These strategies demonstrate that vaccination against *C. albicans* virulence factors not only protects against this fungus itself, thus, preventing pathology at different body sites, but also provide protection against other fungi, and even bacteria. As a result, research into the production of universal fungal vaccines have gained traction in the scientific community, which may not only aid in the discovery of novel fungal vaccine candidates from unexpected and distant species but may also aid in repurposing of some of the effective vaccines used against other pathogens.

## Concluding statement

Informed by mouse studies and fuelled by new techniques, such as single cell RNA sequencing and spatial omics[149, 150], human immunological research is advancing disease treatment while also generating insights into basic immunological concepts[151]. This has led to exciting discoveries regarding the relevance and role of adaptive immunity in immuosurveillance of *C. albicans* commensalism, but also revealed pathological consequences that these responses can have in predisposed patients. However, most work to decipher human immune responses to *C. albicans* has been (understandably) limited to blood. Recently, Lionakis and colleagues used oral mucosal biopsies of APECED patients with CMC to show that tissue-specific immunity is not always reflected by immune responses of circulating cells[34]. Thus, studying tissues directly will aid our understanding of tissue-resident cell types (such as resident memory lymphocytes and tissue-specific stroma) and inform of tissue-specific immune mechanisms driving or preventing pathologies. Accordingly, studying the diverse *C. albicans*-mediated pathologies requires usage of relevant mouse models. For instance, *C. albicans* infections and immune responses should be studied in colonized animals rather than naïve and acutely infected mice. In this line, commensal isolates, which are able to colonize fully immunocompetent animals[20, 84] should be used to closely mimic scenarios seen in patients.

## References

[1] Gouba N & Drancourt M Digestive tract mycobiota: a source of infection. Med. et. maladies infectieuses 45, 9–16 (2015). 10.1016/j.medmal.2015.01.007 25684583

[2] Underhill DM & Pearlman E Immune interactions with pathogenic and commensal fungi: a two-way street. Immunity 43, 845–858 (2015). 26588778 10.1016/j.immuni.2015.10.023 PMC4865256

[3] Wheeler ML Immunological consequences of intestinal fungal dysbiosis. Cell Host Microbe 19, 865–873 (2016). 27237365 10.1016/j.chom.2016.05.003 PMC4900921

[4] Iliev ID & Leonardi I Fungal dysbiosis: immunity and interactions at mucosal barriers. Nat. Rev. Immunol. 17, 635–646 (2017). 28604735 10.1038/nri.2017.55 PMC5724762

[5] Revankar SG, Sobel JD Mucosal Candidiasis. In: Calderone RA, Clancy CJ (eds.) Candida and Candidiasis, 2nd edn. ASM Press.

[6] Pappas PG, Lionakis MS, Arendrup MC, Ostrosky-Zeichner L & Kullberg BJ Invasive candidiasis. Nat. Rev. Dis. Prim. 4, 18026 (2018). 29749387 10.1038/nrdp.2018.26

[7] Li XV, Leonardi I & Iliev ID Gut mycobiota in immunity and inflammatory disease. Immunity 50, 1365–1379 (2019). 31216461 10.1016/j.immuni.2019.05.023 PMC6585451

[8] Lionakis MS & Levitz SM Host control of fungal infections: lessons from basic studies and human cohorts. Annu. Rev. Immunol. 36, 157–191 (2018). 29237128 10.1146/annurev-immunol-042617-053318

[9] Sparber F & LeibundGut-Landmann S Interleukin-17 in antifungal immunity. Pathogens 8, 54 (2019). 10.3390/pathogens8020054 PMC6630750 31013616

[10] Doron I Mycobiota-induced IgA antibodies regulate fungal commensalism in the gut and are dysregulated in Crohn’s disease.Nat. Microbiol. 6, 1493–1504 (2021). 34811531 10.1038/s41564-021-00983-z PMC8622360

[11] Millet N, Solis NV & Swidergall M Mucosal IgA prevents commensal candida albicans dysbiosis in the oral cavity. Front. Immunol. 11, 555363 (2020) 33193324 10.3389/fimmu.2020.555363 PMC7642201

[12] Ost KS Adaptive immunity induces mutualism between commensal eukaryotes. Nature 596, 114–118 (2021). 34262174 10.1038/s41586-021-03722-w PMC8904204

[13] Acosta-Rodriguez EV Surface phenotype and antigenic specificity of human interleukin 17-producing T helper memory cells. Nat. Immunol. 8, 639–646 (2007). 17486092 10.1038/ni1467

[14] Bacher P Human anti-fungal Th17 immunity and pathology rely on cross-reactivity against Candida albicans. Cell 176, 1340–1355 e1315 (2019). 30799037 10.1016/j.cell.2019.01.041

[15] Becattini S T cell immunity. Functional heterogeneity of human memory CD4(+) T cell clones primed by pathogens or vaccines. Science 347, 400–406 (2015). 25477212 10.1126/science.1260668

[16] Park CO Staged development of long-lived T-cell receptor alphabeta TH17 resident memory T-cell population to Candida albicans after skin infection. J. Allergy Clin. Immunol. 142, 647–662 (2018). 29128674 10.1016/j.jaci.2017.09.042 PMC5943196

[17] Conti HR Th17 cells and IL-17 receptor signaling are essential for mucosal host defense against oral candidiasis. J. Exp. Me d. 206, 299–311 (2009 10.1084/jem.20081463 PMC2646568 19204111

[18] Kirchner FR & LeibundGut-Landmann S Tissue-resident memory Th17 cells maintain stable fungal commensalism in the oral mucosa. Mucosal Immunol. 14, 455–467 (2021). 32719409 10.1038/s41385-020-0327-1 PMC7946631

[19] Gladiator A, Wangler N, Trautwein-Weidner K & LeibundGut-Landmann S Cutting edge: IL-17-secreting innate lymphoid cells are essential for host defense against fungal infection. J. Immunol. 190, 521–525 (2013). 23255360 10.4049/jimmunol.1202924

[20] Schonherr FA The intraspecies diversity of C. albicans triggers qualitatively and temporally distinct host responses that deter mine the balance between commensalism and pathogenicity. Mucosal Immunol. 10, 1335–1350 (2017). 28176789 10.1038/mi.2017.2

[21] Verma AH Oral epithelial cells orchestrate innate type 17 responses to Candida albicans through the virulence factor candidalysin. Sci. Immunol 2, eaam8834 (2017). 29101209 10.1126/sciimmunol.aam8834 PMC5881387

[22] Kashem SW Nociceptive sensory fibers drive Interleukin-23 production from CD301b+ dermal dendritic cells and drive protective cutaneous immunity. Immunity 43, 515–526 (2015). 26377898 10.1016/j.immuni.2015.08.016 PMC4607048

[23] Sparber F Langerin+ DCs regulate innate IL-17 production in the oral mucosa during Candida albicans-mediated infection. PLoS Pathog. 14, e1007069 (2018). 29782555 10.1371/journal.ppat.1007069 PMC5983869

[24] Kashem SW Candida albicans morphology and dendritic cell subsets determine T helper cell differentiation. Immunity 42, 356–366 (2015). 25680275 10.1016/j.immuni.2015.01.008 PMC4343045

[25] Igyarto BZ Skin-resident murine dendritic cell subsets promote distinct and opposing antigen-specific T helper cell responses. Immunity 35, 260–272 (2011). 21782478 10.1016/j.immuni.2011.06.005 PMC3163010

[26] Trautwein-Weidner K Antigen-specific Th17 cells are primed by distinct and complementary dendritic cell subsets in oropharyngeal candidiasis. PLoS Pathog. 11, e1005164 (2015). 26431538 10.1371/journal.ppat.1005164 PMC4591991

[27] Korn T, Bettelli E, Oukka M & Kuchroo VK IL-17 and Th17 cells. Annu. Rev. Immunol. 27, 485–517 (2009). 19132915 10.1146/annurev.immunol.021908.132710

[28] Ansaldo E, Farley TK & Belkaid Y Control of immunity by the microbiota. Annu. Rev. Immunol. 39, 449–479 (2021) 33902310 10.1146/annurev-immunol-093019-112348

[29] Omenetti S The intestine harbors functionally distinct homeostatic tissue-resident and inflammatory Th17 cells. Immunity 51, 77–89.e76 (2019) 31229354 10.1016/j.immuni.2019.05.004 PMC6642154

[30] Swarnalekha N T resident helper cells promote humoral responses in the lung. Sci. Immunol. 6, eabb6808 (2021). 33419790 10.1126/sciimmunol.abb6808 PMC8063390

[31] Tamoutounour S Keratinocyte-intrinsic MHCII expression controls microbiota-induced Th1 cell responses. Proc. Natl. Acad. Sci. USA 116, 23643–23652 (2019). 31672911 10.1073/pnas.1912432116 PMC6876208

[32] Leonardi I Mucosal fungi promote gut barrier function and social behavior via Type 17 immunity. Cell 185, 831–846 e814 (2022). 35176228 10.1016/j.cell.2022.01.017 PMC8897247

[33] Markey L Pre-colonization with the commensal fungus Candida albicans reduces murine susceptibility to Clostridium difficile infection. Gut Microbes 9, 497–509 (2018). 29667487 10.1080/19490976.2018.1465158 PMC6287688

[34] Break TJ Aberrant type 1 immunity drives susceptibility to mucosal fungal infections. Science 371, eaay5731 (2021). 33446526 10.1126/science.aay5731 PMC8326743

[35] Shao TY Commensal Candida albicans Positively Calibrates Systemic Th17 Immunological Responses. Cell Host Microbe 25, 404–417.e406 (2019). 30870622 10.1016/j.chom.2019.02.004 PMC6419754

[36] Tso GHW Experimental evolution of a fungal pathogen into a gut symbiont. Science 362, 589–595 (2018). 30385579 10.1126/science.aat0537

[37] Chen C IL-17 is a neuromodulator of Caenorhabditis elegans sensory responses. Nature 542, 43–48 (2017). 28099418 10.1038/nature20818 PMC5503128

[38] Reed MD IL-17a promotes sociability in mouse models of neurodevelopmental disorders. Nature 577, 249–253 (2020). 31853066 10.1038/s41586-019-1843-6 PMC8112727

[39] Choi GB The maternal interleukin-17a pathway in mice promotes autism-like phenotypes in offspring. Science 351, 933–939. (2016) 26822608 10.1126/science.aad0314 PMC4782964

[40] Alves de Lima K Meningeal gammadelta T cells regulate anxiety-like behavior via IL-17a signaling in neurons. Nat. Immunol. 21, 1421–1429 (2020). 32929273 10.1038/s41590-020-0776-4 PMC8496952

[41] Strati F New evidences on the altered gut microbiota in autism spectrum disorders. Microbiome 5, 24 (2017). 28222761 10.1186/s40168-017-0242-1 PMC5320696

[42] Zou R Dysbiosis of gut fungal microbiota in children with autism spectrum disorders. J. Autism Dev. Disord. 51, 267–275 (2021). 32447559 10.1007/s10803-020-04543-y

[43] Kirchner FR Persistence of Candida albicans in the oral mucosa induces a curbed inflammatory host response that is independent of immunosuppression. Front Immunol. 10, 330 (2019). 30873177 10.3389/fimmu.2019.00330 PMC6400982

[44] Huppler AR Role of neutrophils in IL-17-dependent immunity to mucosal candidiasis. J. Immunol. 192, 1745–1752 (2014). 24442441 10.4049/jimmunol.1302265 PMC3946223

[45] Trautwein-Weidner K, Gladiator A, Nur S, Diethelm P & LeibundGut-Landmann S IL-17-mediated antifungal defense in the oral mucosa is independent of neutrophils. Mucosal Immunol. 8, 221–231 (2015). 25005360 10.1038/mi.2014.57

[46] Keppler SJ, Goess MC & Heinze JM The wanderings of gut-derived iga plasma cells: impact on systemic immune responses. Front Immunol. 12, 670290 (2021). 33936114 10.3389/fimmu.2021.670290 PMC8081896

[47] Dimitrov JD & Lacroix-Desmazes S Noncanonical functions of antibodies. Trends Immunol. 41, 379–393 (2020). 32273170 10.1016/j.it.2020.03.006

[48] Janeway C Immunobiology 5: the immune system in health and disease. (Garland Pub., New York, 2001).

[49] Rollenske T & Macpherson AJ Anti-commensal Ig—from enormous diversity to clear function. Mucosal Immunol. 13, 1–2 (2020). 31719642 10.1038/s41385-019-0223-8

[50] Sterlin D, Fadlallah J, Slack E & Gorochov G The antibody/microbiota interface in health and disease. Mucosal Immunol. 13, 3–11 (2020). 31413347 10.1038/s41385-019-0192-y

[51] Romani L Immunity to fungal infections. Nat. Rev. Immunol. 11, 275–288 (2011). 21394104 10.1038/nri2939

[52] Lionakis MS, Iliev ID & Hohl TM Immunity against fungi. JCI Insight 2, 93156 (2017). 28570272 10.1172/jci.insight.93156 PMC5453709

[53] Swidergall M, Solis NV, Lionakis MS & Filler SG EphA2 is an epithelial cell pattern recognition receptor for fungal β-glucans. Nat. Microbiol. 3, 53–61 (2018). 29133884 10.1038/s41564-017-0059-5 PMC5736406

[54] Swidergall M EphA2 is a neutrophil receptor for Candida albicans that stimulates antifungal activity during oropharyngeal infection. Cell Rep. 28, 423–433.e425 (2019). 31291578 10.1016/j.celrep.2019.06.020 PMC6638578

[55] López-Ribot JL, Casanova M, Murgui A & Martínez JP Antibody response to Candida albicans cell wall antigens. FEMS Immunol. Med. Microbiol. 41, 187–196 (2004). 15196567 10.1016/j.femsim.2004.03.012

[56] Roco JA Class-switch recombination occurs infrequently in germinal centers. Immunity 51, 337–350.e337 (2019). 31375460 10.1016/j.immuni.2019.07.001 PMC6914312

[57] Stavnezer J, Guikema JE & Schrader CE Mechanism and regulation of class switch recombination. Annu Rev. Immunol. 26, 261–292 (2008). 18370922 10.1146/annurev.immunol.26.021607.090248 PMC2707252

[58] Doron I Human gut mycobiota tune immunity via CARD9-dependent induction of anti-fungal IgG antibodies. Cell 184, 1017–1031.e1014 (2021). 33548172 10.1016/j.cell.2021.01.016 PMC7936855

[59] Leonardi I CX3CR1(+) mononuclear phagocytes control immunity to intestinal fungi. Science 359, 232–236 (2018). 29326275 10.1126/science.aao1503 PMC5805464

[60] Luo P, Yang Z, Chen B & Zhong X The multifaceted role of CARD9 in inflammatory bowel disease. J. Cell. Mol. Med. 24, 34–39 (2020). 31696662 10.1111/jcmm.14770 PMC6933369

[61] Yamamoto-Furusho JK Caspase recruitment domain (CARD) family (CARD9, CARD10, CARD11, CARD14 and CARD15) are increased during active inflammation in patients with inflammatory bowel disease. J. Inflamm. 15, 13 (2018). 10.1186/s12950-018-0189-4 PMC6042317 30008619

[62] Brand S Increased expression of the chemokine fractalkine in Crohn’s disease and association of the fractalkine receptor T280M polymorphism with a fibrostenosing disease Phenotype. Am. J. Gastroenterol. 101, 99–106 (2006). 16405540 10.1111/j.1572-0241.2005.00361.x

[63] Sabate JM The V249I polymorphism of the CX3CR1 gene is associated with fibrostenotic disease behavior in patients with C rohn’s disease. Eur. J. Gastroenterol. Hepatol. 20, 748–755 (2008). 18617779 10.1097/MEG.0b013e3282f824c9

[64] Drummond RA CARD9-dependent neutrophil recruitment protects against fungal invasion of the central nervous system. PLoS Pathog. 11, e1005293 (2015). 26679537 10.1371/journal.ppat.1005293 PMC4683065

[65] Drummond RA CARD9(+) microglia promote antifungal immunity via IL-1β- and CXCL1-mediated neutrophil recruitment. Nat. Immunol. 20, 559–570 (2019). 30996332 10.1038/s41590-019-0377-2 PMC6494474

[66] Damelang T, Rogerson SJ, Kent SJ & Chung AW Role of IgG3 in infectious diseases. Trends Immunol. 40, 197–211 (2019). 30745265 10.1016/j.it.2019.01.005

[67] Li Y, Jin L & Chen T The effects of secretory IgA in the mucosal immune system. Biomed. Res Int. 2020, 2032057 (2020). 31998782 10.1155/2020/2032057 PMC6970489

[68] Mantis NJ, Rol N & Corthesy B Secretory IgA’s complex roles in immunity and mucosal homeostasis in the gut. Mucosal Immunol. 4, 603–611 (2011). 21975936 10.1038/mi.2011.41 PMC3774538

[69] Mathias A, Pais B, Favre L, Benyacoub J & Corthésy B Role of secretory IgA in the mucosal sensing of commensal bacteria. Gut microbes 5, 688–695 (2014). 25536286 10.4161/19490976.2014.983763 PMC4615909

[70] Moor K High-avidity IgA protects the intestine by enchaining growing bacteria. Nature 544, 498–502 (2017). 28405025 10.1038/nature22058

[71] Wich M Functionality of the human antibody response to Candida albicans. Virulence 12, 3137–3148 (2021). 34923920 10.1080/21505594.2021.2015116 PMC8923069

[72] Mora JR & von Andrian UH Differentiation and homing of IgA-secreting cells. Mucosal Immunol. 1, 96–109 (2008). 19079167 10.1038/mi.2007.14

[73] Hepworth MR, Greenhalgh AD & Cook PC B cells on the brain: meningeal IgA and a novel gut-brain firewall. Immunol. Cell Biol. 99, 17–20 (2021). 33107992 10.1111/imcb.12412

[74] Fitzpatrick Z Gut-educated IgA plasma cells defend the meningeal venous sinuses. Nature 587, 472–476 (2020). 33149302 10.1038/s41586-020-2886-4 PMC7748383

[75] Pabst O & Slack E IgA and the intestinal microbiota: the importance of being specific. Mucosal Immunol. 13, 12–21 (2020). 31740744 10.1038/s41385-019-0227-4 PMC6914667

[76] Fillatreau S B cells and their cytokine activities implications in human diseases. Clin. Immunol. 186, 26–31 (2018). 28736271 10.1016/j.clim.2017.07.020 PMC5844600

[77] Li R Antibody-independent function of human B cells contributes to antifungal T cell responses. J. Immunol. 198, 3245–3254 (2017). 28275140 10.4049/jimmunol.1601572

[78] Ferreira-Gomes M B cell recognition of candida albicans hyphae via TLR 2 promotes IgG1 and IL-6 secretion for T(H)17 differentiation. Front Immunol. 12, 698849 (2021). 34819929 10.3389/fimmu.2021.698849 PMC8606576

[79] Hirakawa MP Genetic and phenotypic intra-species variation in Candida albicans. Genome Res. 25, 413–425 (2015). 25504520 10.1101/gr.174623.114 PMC4352881

[80] Ropars J Gene flow contributes to diversification of the major fungal pathogen Candida albicans. Nat. Commun. 9, 2253 (2018). 29884848 10.1038/s41467-018-04787-4 PMC5993739

[81] Wang JM, Bennett RJ & Anderson MZ The genome of the human pathogen Candida albicans is shaped by mutation and cryptic sexual recombination. mBio 9, e01205–e01218 (2018). 30228236 10.1128/mBio.01205-18 PMC6143739

[82] MacCallum DM Property differences among the four major Candida albicans strain clades. Eukaryot. Cell 8, 373–387 (2009). 19151328 10.1128/EC.00387-08 PMC2653250

[83] Marakalala MJ Differential adaptation of Candida albicans in vivo modulates immune recognition by dectin-1. PLoS Pathog. 9, e1003315 (2013). 23637604 10.1371/journal.ppat.1003315 PMC3630191

[84] McDonough LD Candida albicans Isolates 529L and CHN1 Exhibit Stable Colonization of the Murine Gastrointestinal Tract. mBio 12, e0287821 (2021). 34724818 10.1128/mBio.02878-21 PMC8561340

[85] Fan D Activation of HIF-1alpha and LL-37 by commensal bacteria inhibits Candida albicans colonization. Nat. Med. 21, 808–814 (2015). 26053625 10.1038/nm.3871 PMC4496259

[86] Gerwien F Clinical Candida albicans vaginal isolates and a laboratory strain show divergent behaviors during macrophage interactions. mSphere 5, e00393–00320 (2020). 32817377 10.1128/mSphere.00393-20 PMC7407065

[87] Ballou ER Lactate signalling regulates fungal beta-glucan masking and immune evasion. Nat. Microbiol. 2, 16238 (2016). 27941860 10.1038/nmicrobiol.2016.238 PMC5704895

[88] Mishra PK, Baum M & Carbon J DNA methylation regulates phenotype-dependent transcriptional activity in Candida albicans. Proc. Natl. Acad. Sci. USA 108, 11965–11970 (2011). 21730141 10.1073/pnas.1109631108 PMC3141964

[89] Forche A Rapid phenotypic and genotypic diversification after exposure to the oral host niche in Candida albicans. Genetics 209, 725–741 (2018). 29724862 10.1534/genetics.118.301019 PMC6028260

[90] Forche A Selection of Candida albicans trisomy during oropharyngeal infection results in a commensal-like phenotype. PLoS Genet. 15, e1008137 (2019). 31091232 10.1371/journal.pgen.1008137 PMC6538192

[91] Sitterle E Within-host genomic diversity of Candida albicans in healthy carriers. Sci. Rep. 9, 2563 (2019). 30796326 10.1038/s41598-019-38768-4 PMC6385308

[92] Braunsdorf C & LeibundGut-Landmann S Modulation of the fungal-host interaction by the intra-species diversity of C. albicans. Pathogens 7, 11 (2018). 10.3390/pathogens7010011 PMC5874737 29342100

[93] Hurabielle C Immunity to commensal skin fungi promotes psoriasiform skin inflammation. Proc. Natl. Acad. Sci. USA 117, 16465–16474 (2020). 32601220 10.1073/pnas.2003022117 PMC7368261

[94] Ovcina-Kurtovic N, Kasumagic-Halilovic E, Helppikangans H & Begic J Prevalence of Candida species in patients with psoriasis. Acta Dermatovenerol. Croat 24, 209–213 (2016). 27663922

[95] Crutcher N Oral nystatin in the treatment of psoriasis. Arch. Dermatol. 120, 435–436 (1984). 6703746

[96] Sparber F The Skin Commensal Yeast Malassezia triggers a Type 17 response that coordinates anti-fungal immunity and exacerbates skin inflammation. Cell Host Microbe 25, 389–403.e386 (2019). 30870621 10.1016/j.chom.2019.02.002

[97] Chehoud C Fungal signature in the gut microbiota of pediatric patients with inflammatory bowel disease. Inflamm. Bowel Dis. 21, 1948–1956 (2015). 26083617 10.1097/MIB.0000000000000454 PMC4509842

[98] Hoarau G Bacteriome and mycobiome interactions underscore microbial dysbiosis in familial Crohn’s disease. mBio 7, e01250–01216 (2016). 27651359 10.1128/mBio.01250-16 PMC5030358

[99] Li Q Dysbiosis of gut fungal microbiota is associated with mucosal inflammation in Crohn’s disease. J. Clin. Gastroenterol. 48, 513–523 (2014). 24275714 10.1097/MCG.0000000000000035 PMC4059552

[100] Liguori G Fungal dysbiosis in mucosa-associated microbiota of Crohn’s disease patients. J. Crohns Colitis 10, 296–305 (2016). 26574491 10.1093/ecco-jcc/jjv209 PMC4957473

[101] Sokol H Fungal microbiota dysbiosis in IBD. Gut 66, 1039–1048 (2017). 26843508 10.1136/gutjnl-2015-310746 PMC5532459

[102] Standaert-Vitse A Candida albicans colonization and ASCA in familial Crohn’s disease. Am. J. Gastroenterol. 104, 1745–1753 (2009). 19471251 10.1038/ajg.2009.225

[103] Li XV Immune regulation by fungal strain diversity in inflammatory bowel disease. Nature 603, 672–678 (2022). 35296857 10.1038/s41586-022-04502-w PMC9166917

[104] Fauny M Paradoxical gastrointestinal effects of interleukin-17 blockers. Ann. Rheum. Dis. 79, 1132–1138 (2020). 32719044 10.1136/annrheumdis-2020-217927

[105] Lee JY Serum Amyloid A proteins induce pathogenic Th17 cells and promote inflammatory disease. Cell 180, 79–91.e16 (2020). 31866067 10.1016/j.cell.2019.11.026 PMC7039443

[106] Kumar P Intestinal Interleukin-17 receptor signaling mediates reciprocal control of the gut microbiota and autoimmune inflammation. Immunity 44, 659–671 (2016). 26982366 10.1016/j.immuni.2016.02.007 PMC4794750

[107] Jiang TT Commensal fungi recapitulate the protective benefits of intestinal bacteria. Cell Host Microbe 22, 809–816.e804 (2017). 29174402 10.1016/j.chom.2017.10.013 PMC5730478

[108] Nguyen LD, Viscogliosi E & Delhaes L The lung mycobiome: an emerging field of the human respiratory microbiome. Front Microbiol 6, 89 (2015). 25762987 10.3389/fmicb.2015.00089 PMC4327734

[109] Harrison OJ Commensal-specific T cell plasticity promotes rapid tissue adaptation to injury. Science 2019; 363(6422). 10.1126/science.aat6280 PMC7304459 30523076

[110] Scheffold A, Bacher P & LeibundGut-Landmann S T cell immunity to commensal fungi. Curr. Opin. Microbiol. 58, 116–123 (2020). 33120172 10.1016/j.mib.2020.09.008

[111] Bar E A novel Th cell epitope of Candida albicans mediates protection from fungal infection. J. Immunol. 188, 5636–5643 (2012). 22529294 10.4049/jimmunol.1200594

[112] McKenzie H, Main J, Pennington CR & Parratt D Antibody to selected strains of Saccharomyces cerevisiae (baker’s and brewer’s yeast) and Candida albicans in Crohn’s disease. Gut 31, 536–538 (1990). 2190866 10.1136/gut.31.5.536 PMC1378569

[113] Vermeire S Anti-Saccharomyces cerevisiae antibodies (ASCA), phenotypes of IBD, and intestinal permeability: a study in IBD families. Inflamm. Bowel Dis. 7, 8–15 (2001). 11233666 10.1097/00054725-200102000-00002

[114] Standaert-Vitse A Candida albicans is an immunogen for anti-Saccharomyces cerevisiae antibody markers of Crohn’s disease. Gastroenterology 130, 1764–1775 (2006). 16697740 10.1053/j.gastro.2006.02.009

[115] Schaffer T Anti-Saccharomyces cerevisiae mannan antibodies (ASCA) of Crohn’s patients crossreact with mannan from other yeast strains, and murine ASCA IgM can be experimentally induced with Candida albicans. Inflamm. Bowel Dis 13, 1339–1346 (2007). 17636567 10.1002/ibd.20228

[116] Jawhara S Colonization of mice by Candida albicans is promoted by chemically induced colitis and augments inflammatory responses through Galectin-3. J. Infect. Dis. 197, 972–980 (2008). 18419533 10.1086/528990

[117] Limon JJ Malassezia is associated with Crohn’s disease and exacerbates colitis in mouse models. Cell Host Microbe 25, 377–388.e376 (2019). 30850233 10.1016/j.chom.2019.01.007 PMC6417942

[118] Israeli E Anti-Saccharomyces cerevisiae and antineutrophil cytoplasmic antibodies as predictors of inflammatory bowel disease. Gut 54, 1232–1236 (2005). 16099791 10.1136/gut.2004.060228 PMC1774672

[119] Lewis JD Inflammation, antibiotics, and diet as environmental stressors of the gut microbiome in pediatric Crohn’s disease. Cell Host Microbe 18, 489–500 (2015). 26468751 10.1016/j.chom.2015.09.008 PMC4633303

[120] Hartmann P Dynamic changes of the fungal microbiome in alcohol use disorder. Front Physiol. 12, 699253 (2021). 34349667 10.3389/fphys.2021.699253 PMC8327211

[121] Yang AM Intestinal fungi contribute to development of alcoholic liver disease. J. Clin. Invest. 127, 2829–2841 (2017). 28530644 10.1172/JCI90562 PMC5490775

[122] Lang S Intestinal fungal dysbiosis and systemic immune response to fungi in patients with alcoholic hepatitis. Hepatology 71, 522–538 (2020). 31228214 10.1002/hep.30832 PMC6925657

[123] Demir M The fecal mycobiome in non-alcoholic fatty liver disease. J. Hepatol. 76, 788–799 (2021). 34896404 10.1016/j.jhep.2021.11.029 PMC8981795

[124] Horst AK, Kumashie KG, Neumann K, Diehl L & Tiegs G Antigen presentation, autoantibody production, and therapeutic targets in autoimmune liver disease. Cell Mol. Immunol. 18, 92–111 (2021). 10.1038/s41423-020-00568-6 PMC7852534 33110250

[125] Browne SK & Holland SM Anti-cytokine autoantibodies explain some chronic mucocutaneous candidiasis. Immunol. Cell Biol. 88, 614–615 (2010). 20548324 10.1038/icb.2010.72 PMC4103911

[126] Kisand K Chronic mucocutaneous candidiasis in APECED or thymoma patients correlates with autoimmunity to Th17-associated cytokines. J. Exp. Med. 207, 299–308 (2010). 20123959 10.1084/jem.20091669 PMC2822605

[127] Puel A Autoantibodies against IL-17A, IL-17F, and IL-22 in patients with chronic mucocutaneous candidiasis and autoimmune polyendocrine syndrome type I. J. Exp. Med 207, 291–297 (2010). 20123958 10.1084/jem.20091983 PMC2822614

[128] Ferre EM Redefined clinical features and diagnostic criteria in autoimmune polyendocrinopathy-candidiasis-ectodermal dystrophy. JCI Insight 1, e88782 (2016). 27588307 10.1172/jci.insight.88782 PMC5004733

[129] Orlova EM Expanding the phenotypic and genotypic landscape of autoimmune polyendocrine Syndrome Type 1. J. Clin. Endocrinol. Metab. 102, 3546–3556 (2017). 28911151 10.1210/jc.2017-00139

[130] Break TJ Response to comments on “Aberrant type 1 immunity drives susceptibility to mucosal fungal infections”. Science 373, eabi8835 (2021). 33446526 10.1126/science.aay5731 PMC8326743

[131] Doherty M, Buchy P, Standaert B, Giaquinto C & Prado-Cohrs D Vaccine impact: Benefits for human health. Vaccine 34, 6707–6714 (2016). 27773475 10.1016/j.vaccine.2016.10.025

[132] Bougnoux ME Multilocus sequence typing reveals intrafamilial transmission and microevolutions of Candida albicans isolates from the human digestive tract. J. Clin. Microbiol 44, 1810–1820 (2006). 16672411 10.1128/JCM.44.5.1810-1820.2006 PMC1479199

[133] Tso GHW, Reales-Calderon JA & Pavelka N The elusive Anti-Candida Vaccine: lessons from the past and opportunities for the future. Front Immunol. 9, 897 (2018). 29755472 10.3389/fimmu.2018.00897 PMC5934487

[134] Kullberg BJ & Arendrup MC Invasive Candidiasis. N. Engl. J. Med 373, 1445–1456 (2015). 26444731 10.1056/NEJMra1315399

[135] Swidergall M & Filler SG Oropharyngeal Candidiasis: fungal invasion and epithelial cell responses. PLoS Pathog. 13, e1006056 (2017). 28081240 10.1371/journal.ppat.1006056 PMC5230744

[136] Lin L Th1-Th17 cells mediate protective adaptive immunity against Staphylococcus aureus and Candida albicans infection in mice. PLoS Pathog. 5, e1000703 (2009). 20041174 10.1371/journal.ppat.1000703 PMC2792038

[137] Schmidt CS NDV-3, a recombinant alum-adjuvanted vaccine for Candida and Staphylococcus aureus, is safe and immunogenic in healthy adults. Vaccine 30, 7594–7600 (2012). 23099329 10.1016/j.vaccine.2012.10.038 PMC3513491

[138] Alqarihi A, Singh S, Edwards JE Jr., Ibrahim AS & Uppuluri P NDV-3A vaccination prevents C. albicans colonization of jugular vein catheters in mice. Sci. Rep. 9, 6194 (2019). 30996274 10.1038/s41598-019-42517-y PMC6470131

[139] Ibrahim AS NDV-3 protects mice from vulvovaginal candidiasis through T- and B-cell immune response. Vaccine 31, 5549–5556 (2013). 24063977 10.1016/j.vaccine.2013.09.016 PMC3866209

[140] Spellberg BJ Efficacy of the anti-Candida rAls3p-N or rAls1p-N vaccines against disseminated and mucosal candidiasis. J. Infect. Dis. 194, 256–260 (2006). 16779733 10.1086/504691

[141] Singh S The NDV-3A vaccine protects mice from multidrug resistant Candida auris infection. PLoS Pathog. 15, e1007460 (2019). 31381597 10.1371/journal.ppat.1007460 PMC6695204

[142] Edwards JE Jr. A fungal immunotherapeutic vaccine (NDV-3A) for treatment of recurrent vulvovaginal Candidiasis-A Phase 2 randomized, double-blind, placebo-controlled trial. Clin. Infect. Dis. 66, 1928–1936 (2018). 29697768 10.1093/cid/ciy185 PMC5982716

[143] Cassone A, Boccanera M, Adriani D, Santoni G & De Bernardis F Rats clearing a vaginal infection by Candida albicans acquire specific, antibody-mediated resistance to vaginal reinfection. Infect. Immun. 63, 2619–2624 (1995). 7790077 10.1128/iai.63.7.2619-2624.1995 PMC173351

[144] Schaller M The secreted aspartyl proteinases Sap1 and Sap2 cause tissue damage in an in vitro model of vaginal candidiasis based on reconstituted human vaginal epithelium. Infect. Immun. 71, 3227–3234 (2003). 12761103 10.1128/IAI.71.6.3227-3234.2003 PMC155757

[145] Gropp K The yeast Candida albicans evades human complement attack by secretion of aspartic proteases. Mol. Immunol. 47,465–475 (2009). 19880183 10.1016/j.molimm.2009.08.019

[146] Svoboda E Secreted aspartic protease 2 of Candida albicans inactivates factor H and the macrophage factor H-receptors CR3 (CD11b/CD18) and CR4 (CD11c/CD18). Immunol. Lett. 168, 13–21 (2015). 26306739 10.1016/j.imlet.2015.08.009

[147] De Bernardis F Protective role of antimannan and anti-aspartyl proteinase antibodies in an experimental model of Candida albicans vaginitis in rats. Infect. Immun. 65, 3399–3405 (1997). 9234804 10.1128/iai.65.8.3399-3405.1997 PMC175481

[148] Shukla M, Chandley P & Rohatgi S The Role of B-cells and antibodies against Candida vaccine antigens in invasive candidiasis. Vaccines 9, 1159 (2021). 34696267 10.3390/vaccines9101159 PMC8540628

[149] Huang W, Wang D & Yao YF Understanding the pathogenesis of infectious diseases by single-cell RNA sequencing. Micro. Cell 8, 208–222 (2021). 10.15698/mic2021.09.759 PMC8404151 34527720

[150] Butler D Shotgun transcriptome, spatial omics, and isothermal profiling of SARS-CoV-2 infection reveals unique host responses, viral diversification, and drug interactions. Nat. Commun. 12, 1660 (2021). 33712587 10.1038/s41467-021-21361-7 PMC7954844

[151] Varade J, Magadan S & Gonzalez-Fernandez A Human immunology and immunotherapy: main achievements and challenges. Cell Mol. Immunol. 18, 805–828 (2021). 32879472 10.1038/s41423-020-00530-6 PMC7463107

